# O Papel do Treinamento Físico na Melhoria da Qualidade de Vida para Cardiopatia Congênita Adulta: Revisão Sistemática

**DOI:** 10.36660/abc.20240294

**Published:** 2024-11-22

**Authors:** Tugba Siyah, Naciye Vardar Yagli, Ilker Ertugrul, Hayrettin Hakan Aykan, Melda Saglam

**Affiliations:** 1 Hacettepe University Faculty of Physiotherapy and Rehabilitation Ankara Turquia Hacettepe University, Faculty of Physiotherapy and Rehabilitation, Ankara – Turquia; 2 Hacettepe University Faculty of Medicine Department of Pediatric Cardiology Ankara Turquia Hacettepe University, Faculty of Medicine, Department of Pediatric Cardiology, Ankara – Turquia

**Keywords:** Exercício Físico, Cardiopatias Congênitas, Qualidade de Vida, Treino Aeróbico

## Abstract

**Fundamento:**

As diretrizes atuais orientam a prática de exercícios para a maioria dos pacientes com cardiopatia congênita (CPC). No entanto, a atividade física continua baixa em indivíduos com CPC, com pesquisas limitadas sobre os efeitos dos exercícios em adultos.

**Objetivos:**

O objetivo deste estudo é avaliar a segurança e a eficácia do treinamento físico sobre a capacidade de realização do exercício e a qualidade de vida para pacientes com cardiopatia congênita adulta (CPCA).

**Métodos:**

Pesquisamos as bases de dado da PubMed/Medline, Cochrane Library, Web of Science e Scopus até dezembro de 2022 em busca de estudos clínicos randomizados que avaliassem os efeitos do treinamento aeróbico e de resistência sobre a capacidade de realização de exercícios e a qualidade de vida na CPCA. Das 3.517 citações obtidas, dez artigos elegíveis foram incluídos.

**Resultados:**

A metanálise dos estudos clínicos randomizados incluídos (286 participantes) não revelou mudanças significativas no consumo de máximo de oxigênio ou na qualidade de vida na CPCA com treinamento com exercícios (diferença média combinada = 0,33 ml/kg/min [IC de 95%, -0,88 a 1,54 ml/kg/min]; p = 0,60; I^2^ = 3%). No entanto, o aumento na carga de trabalho máxima foi significativo (diferença média combinada = 8,86 watts [IC de 95%, 0,78 a 16,93], p = 0,03, I^2^ = 0%).

**Conclusões:**

Nossa revisão confirma que o treinamento com exercícios aumenta a carga de trabalho máxima em pacientes com CPCA. No entanto, a falta de um protocolo padronizado entre as intervenções de exercícios nessa população pode ter contribuído para a ausência de uma mudança significativa no pico de VO_2_ e na qualidade de vida observada nos estudos conduzidos. Além disso, a heterogeneidade dos programas de exercícios pode ser um fator contribuinte para a inconsistência dos resultados. Neste contexto, a implementação de protocolos de exercícios padronizados em pesquisas futuras, particularmente com tamanhos de amostra maiores, é fundamental para melhorar a comparabilidade dos resultados. Estudos clínicos randomizados bem projetados, que avaliem o treinamento com exercícios estruturados em pacientes com CPCA, fornecerão dados mais claros.

## Graphical abstract

**Figura Central:**
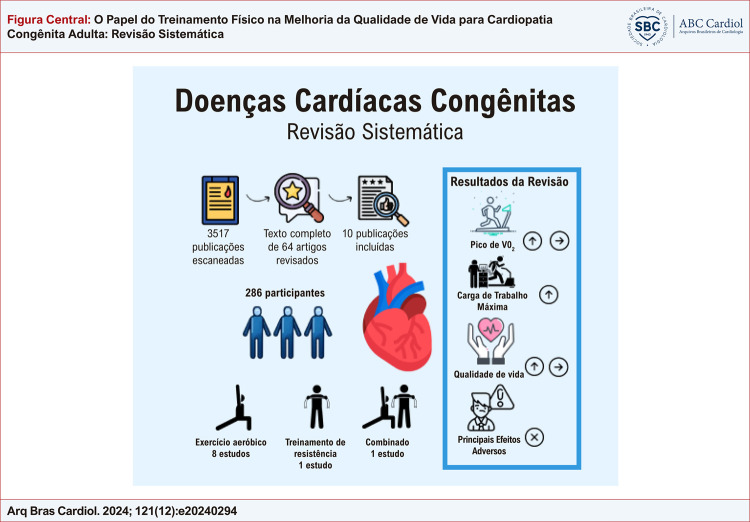
: O Papel do Treinamento Físico na Melhoria da Qualidade de Vida para Cardiopatia Congênita Adulta: Revisão Sistemática

## Introdução

Embora as possibilidades diagnósticas e terapêuticas para cardiopatia congênita (CPC) tenham melhorado significativamente nos últimos anos, a CPC ainda é uma das principais causas de defeitos congênitos, com 13,3 milhões de pacientes em todo o mundo em 2019.^1,2^ Avanços em cardiologia intervencionista, cirurgia cardíaca congênita e no tratamento da CPC reduziram significativamente a mortalidade e aumentaram a população de pacientes com CPC que atingem a idade adulta.^3^ Esses desenvolvimentos rapidamente tornaram a CPC uma condição passível de acompanhar o paciente por toda sua vida.^4^ Espera-se que o número de adultos com CPC aumente em 5% ao ano.^5^ Com o aumento da sobrevida de pacientes com cardiopatia congênita adulta (CPCA), as variáveis de saúde funcional se tornaram um elemento importante na avaliação dos efeitos do tratamento. No passado, o tratamento da CPC era quase inteiramente domínio da cardiologia pediátrica, mas agora deve ser contínuo em todos os sistemas de saúde pediátricos e adultos.^4^ A crescente população com CPCA aumentou a importância das oportunidades de tratamento e tratamento na idade adulta. Sendo assim, pesquisas estão em andamento para atender às várias necessidades da população com CPCA, a fim de facilitar o tratamento da doença e desenvolver diretrizes relevantes.

A intolerância ao exercício é um grande problema para pacientes com CPCA, afetando significativamente sua qualidade de vida. A maioria dos estudos anteriores foi conduzida com pacientes pediátricos e adultos, com uma parcela mínima incluindo apenas adultos. Não existe uma revisão sistemática na literatura que inclua estudos focados apenas em adultos. Portanto, uma revisão sistemática examinando os efeitos do treinamento físico sobre a capacidade de realização de exercício e a qualidade de vida em pacientes com CPCA pode ser benéfica na prática clínica. O objetivo desta revisão foi examinar estudos clínicos randomizados que avaliaram a eficácia e a segurança de programas de treinamento aeróbico e de resistência em paciente com CPCA.

## Métodos

Esta revisão sistemática foi conduzida e relatada de acordo com as diretrizes PRISMA. O protocolo de revisão foi registrado no PROSPERO (Registro Prospectivo Internacional de Revisões Sistemáticas; número de registro CRD42022380143).

### Critério de elegibilidade

Estudos clínicos randomizados, que investigaram os efeitos do treinamento com exercícios aeróbicos e de resistência em pacientes com CPCA foram incluídos nesta revisão sistemática. Os critérios de elegibilidade foram: estudos que incluem apenas indivíduos com CPC maiores de 18 anos; estudos envolvendo o treinamento com exercícios aeróbicos, treinamento de força ou uma combinação de ambos os tipos de treinamento com exercícios. Estudos envolvendo exercícios respiratórios, oxigenoterapia e técnicas motivacionais foram excluídos. Os desfechos primários foram capacidade de realização de exercício (pico de consumo de oxigênio [pico de VO_2_]), carga de trabalho máxima e qualidade de vida.

A pesquisa incluiu estudos publicados em inglês, no período entre janeiro de 1980 e dezembro de 2022, nas bases de dados da PubMed/Medline, Cochrane Central Register of Controlled Trials, Web of Science e Scopus. As palavras-chave foram verificadas no MeSH (Medical Subject Headings) para possibilitar uma estratégia de busca que foi então usada para a obtenção de títulos e resumos de estudos potencialmente relevantes. Dois revisores independentes avaliaram o texto completo dos artigos selecionados para verificar o atendimento aos critérios de elegibilidade. Um formulário de dados foi usado para registrar dados dos artigos examinados. Os autores foram contatados por e-mail quando qualquer confirmação de dados ou informações adicionais foram necessárias.

Características do estudo (por exemplo, ano de publicação, desenho do estudo), características do paciente (por exemplo, idade, diagnóstico), dados de intervenção de exercício (por exemplo, aeróbico, de resistência, combinado) e dados de resultados (por exemplo, incidência de eventos adversos, pico de VO_2_, carga de trabalho máxima, qualidade de vida) foram extraídos dos estudos incluídos.

### Qualidade da evidência

A escala PEDro foi usada para avaliar a qualidade da evidência para esta revisão sistemática. A escala PEDro avalia a qualidade metodológica do estudo, com itens como critérios de inclusão/exclusão, alocação de grupo e cegamento.^6^

Dos estudos clínicos randomizados incluídos, sete especificaram como a randomização foi realizada. Os métodos de randomização usados nos estudos incluíram uma sequência de alocação gerada por computador, randomização com envelopes lacrados e randomização em bloco. Devido à natureza das intervenções, não foi possível cegar os participantes e a equipe. No entanto, as medições de resultados foram cegas em three estudos. Todos os estudos forneceram informações sobre dados de resultados ausentes. Nenhum viés foi observado para relatórios seletivos em qualquer um dos estudos.

### Avaliação de risco de viés

Dois revisores independentes avaliaram o risco de viés nos estudos elegíveis usando a ferramenta Review Manager 5.4 (Cochrane, Reino Unido) para estudos clínicos randomizados.

### Análise estatística

Para a análise estatística, dados de pico de VO_2_, qualidade de vida e carga de trabalho máxima foram registrados. A análise estatística consistiu em comparações de pico de VO_2_, qualidade de vida e carga de trabalho máxima entre os grupos de exercício e controle (sem exercício, cuidados habituais) após a intervenção. Diferenças médias (DMs) agrupadas com IC de 95% e valores de p foram obtidos usando um modelo de efeitos aleatórios (DerSimonian-Laird). Um valor de p de 0,05 foi considerado significativo. Valores de I^2^ entre 25% e 50% foram aceitos como um indicador de heterogeneidade moderada. Valores de I^2^ acima de 50% foram aceitos como um indicador de alta heterogeneidade. O Review Manager 5.4 (Cochrane, Reino Unido) foi usado para a análise dos dados extraídos.^7^

## Resultados

Um total de 3517 publicações foram inicialmente rastreadas, e 83 publicações que atendiam aos critérios de elegibilidade foram revisadas. Dezenove publicações foram excluídas devido a duplicação. O texto completo de 64 artigos foi revisado. Destes, 54 publicações foram excluídas, e 10 publicações foram incluídas no estudo (Figura Central). O tamanho mínimo da amostra foi 10 e o máximo foi 40. A idade média dos participantes variou de 29 a 41 anos.

Oito dos 10 estudos envolveram treinamento com exercícios aeróbicos. O estudo de Avila et al. envolveu treinamento combinado com exercícios aeróbicos e resistência,^8^ e a intervenção conduzida por Cordina et al. envolveu apenas treinamento de resistência. Os estudos incluídos foram publicados a partir de 2003 e aumentaram em número nos últimos 10 anos.^9^ Todos os grupos de controle receberam tratamento padrão. As características dos estudos incluídos são mostradas na Tabela 1.

**Tabela 1:** – Características dos estudos incluídos

Referência	Ano de publicação	Diagnóstico do paciente	Idade do paciente (anos)	Intervenção	Controle	Medições de desfecho	Eventos adversos
Ávila et al.^8^	2016	TF	35 ± 11	Treinamento aeróbico e de resistência combinados	Cuidados habituais (com orientação sobre exercícios)	Pico de VO_2_ (ml/kg/min) PNC (pg/ml) Frequência cardíaca de repouso Equivalentes metabólicos (MET) Duração do exercício	Tontura leve durante o treinamento físico em 2 pacientes
Westhoff-Bleck et al.^15^	2013	TGA	29,3 ± 3,4	Exercício aeróbico	Cuidados habituais	VO_2_ de pico (ml/kg/min) Escala de classificação de esforço percebido de Borg Duração do exercício (min) Carga máxima de trabalho (W) Questionário de cardiomiopatia de Kansas City	Nenhum evento adverso
Winter et al.^16^	2013	Ventrículo direito sistêmico, TGA	32 ± 11	Exercício aeróbico	Cuidados habituais	VO_2_ de pico (ml/kg/min) NT-proBNP (ng/l) Item do Short Form 36 (SF-36)	Nenhum evento adverso
Novaković et al.^10^	2018	TF	39 ± 9	Exercício aeróbico	Cuidados habituais (sem exercício supervisionado)	Pico de VO_2_ (ml/kg/min) Carga máxima de trabalho (W) Equivalentes metabólicos (MET) NT-proBNP (ng/l) Item do Short Form 36 (SF-36) sobre Qualidade de Vida em adultos (CHD-TAAQOL)	Nenhum evento adverso
Sandberg et al.^13^	2018	Doença cardíaca congênita complexa (atresia pulmonar, ToF, TGA)	29 ± 10	Exercício aeróbico	Cuidados habituais	Pico de VO_2_ (ml/kg/min) Carga máxima de trabalho (W) Escala visual analógica EuroQol	Em um caso, o treinamento físico foi interrompido devido ao desconforto do paciente durante a sessão de treinamento físico e possível arritmia
van Dissel et al.^14^	2019	ToF, TGA, Fontan, atresia pulmonar	40 ± 12	Exercício aeróbico	Cuidados habituais	Pico de VO_2_ (ml/kg/min) Limiar anaeróbico (inclinação VE'/V'CO_2_) Carga máxima de trabalho (W) NT-proBNP (ng/l) Item do Short Form 36 (SF-36) Qualidade de vida em adultos (CHD-TAAQOL)	Nenhum evento adverso
Opotowsky et al.^11^	2010	ToF com estenose ou atresia pulmonar ou ventrículo direito de dupla saída	41,1 ± 12,1	Exercício aeróbico	Cuidados habituais	Pico de VO_2_ (ml/kg/min) Carga máxima de trabalho (W) Limiar anaeróbico (inclinação VE'/V'CO_2_) NT-proBNP (ng/l)	Nenhum evento adverso
Rakhmawati et al.^17^	2020	DSA-HAP não corrigido	37,5 ± 8,8 vs. 35,5 ± 10,4	Exercício aeróbico	Cuidados habituais (os indivíduos mantiveram atividades e estilo de vida normais)	Distância do TC6M (m) NT-proBNP (ng/l) Escala visual analógica EuroQol	Nenhum evento adverso
Therrien et al.^12^	2003	TF	35 ± 9,5 vs. 43,3 ± 73	Exercício aeróbico	Cuidados habituais	Pico de VO_2_ (ml/kg/min) Limiar anaeróbico (inclinação VE'/V'CO_2_)	Batimentos ventriculares e atriais prematuros ocasionais em quatro pacientes durante o teste de exercício
Cordina et al.^9^	2012	Fontan	32 ± 2	Treinamento de resistência	Estilo de vida habitual	Pico de VO_2_ (ml/kg/min) Força muscular	Nenhum evento adverso

ToF: Tetralogia de Fallot; TGA: transposição das grandes artérias; DAS: defeito do septo atrial; HAP: hipertensão arterial pulmonar; Pico de VO_2_: consumo máximo de oxigênio; PNC - Peptídeo natriurético cerebral; TC6M: teste de caminhada de 6 minutos.

As intervenções de treinamento com exercícios variaram de 10 a 24 semanas de duração. A frequência mais comum de treinamento com exercícios foi de três vezes por semana, e a duração das sessões de exercícios foi entre 30 e 60 minutos. A frequência cardíaca máxima (FCmáx) e o pico de VO_2_ foram os métodos de preferência para determinar a intensidade do exercício nos estudos incluídos. A intensidade do exercício variou de 60-80% da FCmáx e 50-85% do pico de VO_2_. Os detalhes relativos às intervenções nos relatórios incluídos são mostrados na Tabela 2.

**Tabela 2:** – Características das intervenções de treinamento físico incluídas na revisão

Referência	Tipo	Duração do treinamento (semanas)	Duração da sessão (minutos)	Sessões por semana	Intensidade
Ávila et al.^8^	Treinamento aeróbico e de resistência combinados (corrida, remo, natação e exercícios de sustentação de peso)	12	60	1-2	70-80% FCmáx
Westhoff-Bleck et al.^15^	Cicloergômetro	24	10-30	3-5	50% do pico de VO_2_
Winter et al.^16^	“Step” aeróbico	10	42	3	75-90% FCmáx
Novaković et al.^10^	Ciclismo e/ou caminhada rápida	12	42	2-3	60-80% FCmáx
Sandberg et al.^13^	Cicloergômetro	12	42	3	FC alvo em 75-80% da intensidade (método de Karvonen) ou classificação de Borg de esforço percebido 15-16
van Dissel et al.^14^	Esportes de preferência do paciente (remo, ciclismo e patinação no gelo)	24	45	3	80% da FCmáx
Opotowsky et al.^11^	Esteira, ciclismo ou remo	12	60	2	De acordo com a resposta do participante ao exercício e a taxa de esforço percebido
Rakhmawati et al.^17^	Esteira e caminhada	12	30	3	60-70% FCmáx
Therrien et al.^12^	Cicloergômetro e esteira	12	30-50	3	60-85% do pico de VO_2_
Cordina et al.^9^	Supino, puxada alta (latíssimo do dorso), “leg press”, extensão de joelho, flexão de joelho, elevação de panturrilha no hack e elevação de panturrilha sentado	20	60	3	80% de uma repetição máxima

FCmáx: frequência cardíaca máxima; Pico de VO_2_: consumo máximo de oxigênio. Referências

### Desfechos

#### Pico de VO2

**Figura 1:**
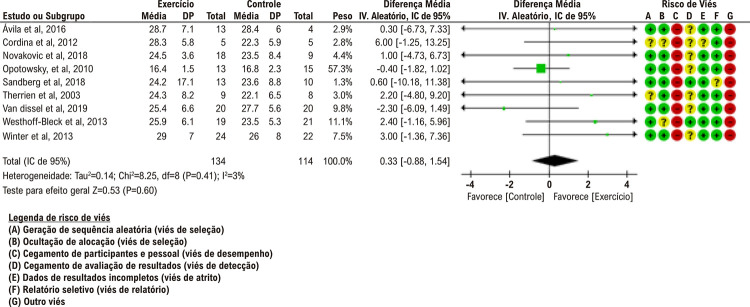
– Gráfico de floresta comparando o treinamento com exercícios vs. controle; resultado: carga de trabalho máxima (W).

Pacientes com CPCA que receberam as intervenções de treinamento com exercícios apresentaram pico de VO_2_ semelhante àqueles que não receberam treinamento com exercícios. Em 8 estudos, o teste de exercício cardiopulmonar (TECP) com carga de trabalho crescente foi realizado no cicloergômetro. Apenas um estudo realizou o TECP na esteira. A análise não mostrou diferenças estatisticamente significativas na melhora do pico de VO_2_ em pacientes com CPCA entre os grupos de intervenção e controle nos estudos clínicos randomizados incluídos (9 estudos, n = 248: DM agrupada: 0,33 ml/kg/min [IC de 95%: −0,88-1,54 ml/kg/min]; p = 0,60; I^2^ = 3%) (Figura 1).^8-16^ Ávila et al. revelaram que o treinamento com exercícios aumentou o pico de VO_2_ em comparação com as medições basais, mas não houve diferença entre o grupo de exercícios e o grupo controle (grupo de intervenção: mediana 27,1 [intervalo interquartil (IIQ): 25,0-33,6] ml/kg/min vs. grupo controle: 29 [IIQ: 25,5-31,0] ml/kg/min, p > 0,05).^8^ Cordina et al. associaram o treinamento com exercícios a um aumento significativo no pico de VO_2_ (Δ 183 ± 31 vs. 5 ± 39 ml/min, p = 0,02).^9^ Novaković *et al*. compararam o treinamento intervalado e o treinamento contínuo com um grupo controle e observaram que o treinamento intervalado melhorou o pico de VO_2_, enquanto o treinamento contínuo não. Não houve diferença significativa nos valores de pico de VO_2_ entre os grupos após o treinamento (grupo de intervalo: mediana 22,9 [IIQ: 21,1-35,4] ml/kg/min, grupo contínuo: 23,6 [IIQ: 20,7-27,8] ml/kg/min e grupo controle: 21,5 [IIQ: 19.4-29.1] ml/kg/min).^10^ Semelhante a outros estudos, Opotowsky et al. revelaram que o valor de pico de VO_2_ melhorou mais no grupo de exercícios em comparação ao grupo controle (Δ 2,2 ml/kg/min, p = 0,002). No entanto, a diferença entre os grupos após o treinamento com exercícios não foi significativa (grupo de intervenção: 16,4 ± 1,5 ml/kg/min vs. grupo controle: 16,8 ± 2,3 ml/kg/min, p > 0,05).^11^

#### Carga de trabalho máxima

**Figura 2:**
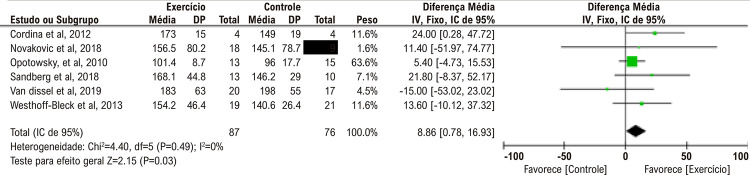
– Gráfico de floresta comparando o treinamento com exercícios vs. controle; resultado: carga de trabalho máxima (W).

A carga de trabalho máxima (em watts) foi medida em 6 dos estudos clínicos randomizados incluídos (total n = 87 no grupo de intervenção e n = 76 no grupo controle). Entre esses estudos, as cargas de trabalho máximas dos participantes aumentaram significativamente após o treinamento com exercícios (6 estudos: DM agrupada: 8,86 [IC de 95%: 0,78-16,93] W, p = 0,03, I^2^ = 0%) (Figura 2).^9-11,13-15^ Cordina *et al*. relataram resultados de carga de trabalho observados após 12 meses de treinamento de resistência de alta intensidade (intervenção vs. grupo controle: 173 ± 15 vs. 149 ± 19 W, respectivamente, p = 0,01).^9^ Na comparação que realizaram entre treinamento intervalado, treinamento contínuo e grupo controle, Novaković et al. descobriram que tanto o treinamento intervalado quanto o contínuo aumentaram a carga de trabalho máxima em comparação com as medições da linha de base (grupo de intervalo: média Δ 9 W, p = 0,002; grupo contínuo: média Δ 15 W, p = 0,003), mas não houve diferença entre os grupos.^10^ Opotowsky et al. observaram uma melhora não significativa na taxa de trabalho de pico (Δ 8,1 W; p = 0,13, Δ 9,2 W ajustado por idade; p = 0,16).^11^ No estudo de Sandberg et al., a carga de trabalho de pico pós-intervenção no TECP incremental foi maior no grupo de intervenção em comparação aos controles (mediana 170 [intervalo: 90-240] vs. 140 [intervalo: 110–200] W, respectivamente) e a mudança na carga de trabalho foi significativamente maior para o grupo de intervenção (média Δ 20 [intervalo: –10-70] vs. Δ 0 [intervalo: –20-15] W, respectivamente, p = 0,003).^13^ Da mesma forma, Westhoff-Bleck et al. observaram que a carga de trabalho máxima aumentou com o treinamento com exercícios (grupo de exercícios: 154,2 ± 46,4 W vs. grupo controle: 140,6 ± 26,4 W).^15^ Ao contrário dos resultados de outros estudos, van Dissel et al. relataram que a carga de trabalho máxima foi menor no grupo de treinamento com exercícios em comparação com o grupo controle. A razão para esse resultado pode ser que a carga máxima de trabalho do grupo controle foi maior antes do treinamento (grupo de exercícios: 183 ± 63 W vs. grupo controle: 198 ± 55 W, p = 0,209).^14^

#### Qualidade de vida

**Figura 3:**
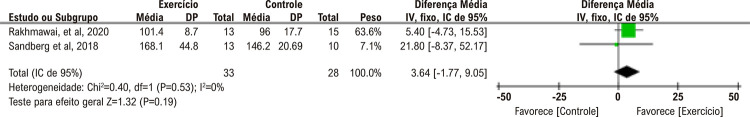
–Gráfico de floresta comparando o treinamento com exercícios vs. controle; resultado: qualidade de vida (EuroQoL EQ-VAS).

A qualidade de vida foi avaliada usando o *Kansas City Cardiomyopathy Questionnaire*, o *36-Item Short Form Health Survey* (SF-36), traduções holandesas e italianas do *CPC TNO/AZL Adult Quality of Life* (CHD-TAAQOL) *Questionnaire, EuroQoL Vertical Visual Analogue Scale* (EQ-VAS) e *Minnesota Living with Heart Failure Questionnaire* (MLHFQ). Os questionários mais frequentemente usados foram o EQ-VAS e o SF-36. Os participantes não mostraram diferença significativa nos escores do EQ-VAS após o treinamento com exercícios (2 estudos: DM: 3,64 pontos [IC de 95%: −1,77-9,05 pontos]; p = 0,19; I^2^= 0%) (Figura 3).^13,16,17^ Em um desses estudos, os escores do EQ-VAS no grupo de exercícios melhoraram incrementalmente da linha de base até a semana 12 (média Δ 15,5, p < 0,001).^17^ Em contraste, Sandberg *et al*. não relataram diferenças significativas na qualidade de vida entre os grupos de estudo (grupo de exercícios: média Δ 0 [intervalo: –21,0-25,0], grupo controle: média Δ 0 [intervalo: –55,0-9,0], p = 0,42), resultado atribuído à alta qualidade de vida na população do estudo antes da intervenção. Três estudos avaliaram a qualidade de vida com o SF-36, mas dados de apenas um estudo estavam disponíveis.^13^ Novaković et al. observaram que apenas o componente mental do questionário SF-36 melhorou em comparação com a linha de base no grupo de treinamento com exercícios contínuos (média Δ 7, p = 0,028). No entanto, não houve outras diferenças significativas dentro ou entre os grupos de intervalo, contínuo e controle.^10^

#### Efeitos adversos

Nenhum dos estudos relatou eventos adversos importantes relacionados ao exercício durante o teste de exercício ou treinamento. Therrien et al. relataram que batimentos ventriculares e atriais prematuros ocasionais foram registrados em quatro pacientes durante o teste de exercício (dois pacientes na linha de base e dois na avaliação de acompanhamento).^12^No estudo de Sandberg et al., o treinamento com exercícios foi interrompido devido ao desconforto do paciente e possível arritmia durante a sessão em um caso, mas nenhuma arritmia foi detectada no teste de exercício subsequente ou na gravação do Holter.^13^ Tontura leve foi relatada durante o treinamento com exercícios em dois dos participantes em outro estudo.^8^

## Discussão

Os resultados desta revisão sistemática de estudos clínicos randomizados sobre intervenções de exercício físico para pacientes com CPCA mostraram que o treinamento com exercícios aumentou a carga de trabalho máxima. No entanto, mais estudos clínicos randomizados são necessários para demonstrar os efeitos sobre a capacidade de realização de exercícios, conforme avaliado pelo pico de VO_2_ e a qualidade de vida (avaliada por EQ-VAS e SF-36). Nenhum evento adverso importante foi relatado durante a intervenção de exercício em nenhum dos estudos incluídos, indicando que o treinamento com exercícios é seguro para a população com CPCA. Esta revisão sistemática inclui o primeiro estudo a investigar o impacto do treinamento físico exclusivamente dentro de pesquisas focadas na população adulta.

Dos estudos incluídos nesta revisão sistemática, sete demonstraram que o treinamento com exercícios aeróbicos contribuiu para melhorias no pico de VO_2_, enquanto apenas dois estudos que avaliaram o pico de VO_2_ não mostraram qualquer melhora com o treinamento com exercícios aeróbicos. A CPCA abrange uma ampla variedade de doenças, e os estudos revisados incluíram pacientes com diversas patologias diferentes, como Tetralogia de Fallot, Transposição das Grandes Artérias, Atresia Pulmonar de Fontan e Defeito do Septo Atrial. A inclusão dessas condições, que envolvem diferentes patologias, resulta na formação de grupos heterogêneos. Sabe-se que o pico de VO_2_ é afetado por diversos fatores, incluindo oxigenação muscular, capacidade de transporte de oxigênio, função endotelial, volume expiratório forçado no primeiro segundo (VEF_1_), capacidade de difusão do pulmão para monóxido de carbono (D_LCO_) e massa muscular.^18,19^ Sendo assim, os resultados inconsistentes e a ausência geral de uma diferença significativa no pico de VO_2_ com o treinamento com exercícios aeróbicos podem ser atribuídos aos grupos de estudo heterogêneos e à influência de múltiplos fatores sobre o pico de VO_2_. Os valores basais de pico de VO_2_ também impactam os resultados pós-exercício. Embora tenha havido uma mudança no pico de VO_2_ com o treinamento físico, valores basais mais baixos no grupo de treinamento físico em comparação com o grupo controle podem levar a uma interpretação errônea dos resultados. Além disso, um tamanho de amostra insuficiente foi observado em vários estudos. Essa situação, somada aos altos desvios-padrão, podem ser fatores que afetaram os resultados. Além disso, embora esteira e ciclismo tenham sido mais comumente usados, as intervenções de exercícios nos estudos incluídos foram bastante heterogêneas. Ávila et al. incluíram exercícios de corrida, remo, natação e levantamento de peso em sua intervenção combinada de treinamento físico, enquanto van Dissel et al. usaram exercícios de remo, ciclismo e patinação no gelo, de acordo com as preferências dos pacientes. Considerando que as respostas de diferentes patologias ao exercício serão diferentes em estudos envolvendo diferentes CPCs, o volume de exercícios pode ter sido insuficiente para adultos.^14^ Outra revisão sistemática recente e metanálise de estudos envolvendo pacientes com tetralogia de Fallot avaliou o efeito do treinamento físico sobre o pico de VO_2_.^20^ Embora essa metanálise tenha incluído indivíduos com CPCA, a idade média dos pacientes no estudo foi de 7,4 a 43,3 anos. A ampla faixa etária pode dificultar a interpretação dos resultados para as populações pediátrica e adulta.

Outro fator importante é o papel da idade na determinação da intensidade e do tipo de exercício apropriados. Incluir volumes de exercício heterogêneos em estudos impede uma compreensão clara do efeito do treinamento com exercícios sobre o pico de VO_2_. O pico de VO_2_ aumenta proporcionalmente ao aumento do nível de exercício ou carga de trabalho. Mais energia por unidade de tempo (potência) é necessária para executar uma carga de trabalho mais pesada. Portanto, em cargas de trabalho maiores, o VO_2_ aumenta proporcionalmente.^21^ De acordo com os resultados dos estudos examinados, um aumento significativo na carga de trabalho foi observado com o treinamento com exercícios aeróbicos.

Nesta revisão, observamos que a qualidade de vida relacionada à saúde após a intervenção do treinamento com exercícios foi semelhante nos grupos de intervenção e controle. De forma semelhante, outra revisão concluiu que qualquer tipo de intervenção de atividade física (promoção de atividade física, treinamento com exercícios e treinamento muscular inspiratório) exerceu pouco ou nenhum efeito sobre a qualidade de vida relacionada à saúde em comparação com o tratamento padrão.^22^ Um estudo indicou que um programa de atividade física cardíaca domiciliar, de intensidade moderada a alta, por 12 semanas, era seguro e viável para crianças com circulação de Fontan e estava associado a uma melhora significativa na qualidade de vida relacionada à saúde relatada pelos pais, mas o programa não melhorou significativamente a qualidade de vida relacionada à saúde autorrelatada pelos pacientes pediátricos.^23^ Em contraste, Rhodes et al. observaram melhorias nos escores do questionário de qualidade de vida relacionado à saúde em 15 pacientes com CPC complexa após um programa de reabilitação cardíaca hospitalar de 12 semanas e revelaram que essa melhora foi sustentada no acompanhamento de 1 ano.^24^ Não podemos esquecer que, em estudos conduzidos na população pediátrica, as famílias têm alta determinação, o que pode afetar os resultados do treinamento. Portanto, estudos com uma faixa etária específica incluindo crianças têm uma generalização muito limitada para a população em geral. Para examinar o efeito do treinamento físico sobre a qualidade de vida relacionada à saúde na população com CPCA, é essencial avaliar a qualidade de vida relacionada à saúde apenas em adultos e observar os resultados do manejo da doença com acompanhamento de longo prazo. A qualidade de vida pode ser influenciada por diversos fatores, como bem-estar físico, situação financeira, vida social e estado psicológico e emocional.^25^ Por esse motivo, seria mais apropriado examinar dados de mais estudos, com análises adicionais para entender o efeito do treinamento físico sobre a qualidade de vida. Além disso, em vez de questionários gerais de qualidade de vida, instrumentos desenvolvidos especificamente para CPC podem ser ferramentas de avaliação mais adequadas.

A disfunção musculoesquelética é conhecida por ser relativamente comum em pessoas com CPCA.^26^ Sandberg et al. mostraram que adultos com lesões cardíacas congênitas complexas tinham função muscular prejudicada em comparação com pacientes com lesões simples e controles. Além disso, a complexidade da lesão cardíaca é um fator determinante importante da função muscular dos membros.^27^ Terapias direcionadas à melhora da fraqueza muscular são recomendadas, uma vez que as limitações nos exercícios demonstraram ser fortes preditores de sobrevivência na população com CPC.^28^ A literatura carece de dados referentes à avaliação da força muscular e treinamento de força em adultos com CPC. Acreditamos que mais estudos nesta área serão importantes porque a força muscular é multifatorial e pessoas com CPCA apresentam problemas musculares.^26,29^

Nenhum evento adverso importante relacionado ao exercício foi relatado nos estudos. Tradicionalmente, as preocupações sobre a segurança do treinamento físico resultaram em muitos pacientes com CPC sendo orientados por seus médicos a não praticar esportes ou outras atividades físicas no início da vida, levando à superproteção parental ou ambiental e a restrições indevidas desde a infância.^30^

Indivíduos com CPC têm menor capacidade de realização de exercício em comparação com controles saudáveis.^31^ Esta situação aumenta a importância da reabilitação cardíaca em pacientes com CPC. Foi sugerido que a inclusão da reabilitação cardíaca e do treinamento com exercícios no manejo da doença pode ser benéfica para pacientes com CPCA e intolerância ao exercício.^4^ Embora muitos estudos tenham demonstrado os benefícios do treinamento com exercícios, foi relatado que apenas 19% das pessoas com CPC recebem orientação formal sobre a prática de atividades físicas.^32^ Isso constitui um fator de risco para doença cardiovascular na vida adulta.^33^ Portanto, a prática de exercícios deve ser fortemente encorajada em pacientes com CPC sem restrições físicas.^34^ O fato de que a população com CPCA tem aumentado e até mesmo superado a população pediátrica com CPC exige uma perspectiva sobre o manejo da doença e o planejamento de programas de tratamento para atender às necessidades dessa população.

Um pequeno mas crescente corpo de literatura sugere que, além da terapia médica de rotina, pacientes com CPC clinicamente estáveis devem receber prescrição de um programa de educação de exercícios individualizado, compatível com as características de sua condição após uma avaliação adequada.^35^

### Limitações

A presente revisão sistemática possui algumas limitações. Uma delas é que havia poucos estudos elegíveis avaliando exercícios de resistência e exercícios combinados como intervenções e usando a capacidade de realização de exercício e a qualidade de vida como medidas de resultado. Além disso, os estudos incluídos não apresentaram dados de acompanhamento de longo prazo, o que contribuiria para o manejo da doença após a intervenção do exercício.

## Conclusões

Esta revisão sistemática mostra que a carga máxima de trabalho aumenta com o treinamento com exercícios na população com CPCA. Além disso, mostra que há pouca evidência sob a forma de estudos randomizados para fundamentar a ideia de que os exercícios melhoram a capacidade de realização de exercícios e a qualidade de vida na CPCA. Portanto, estudos clínicos randomizados bem projetados de treinamento com exercícios padronizados, incluindo treinamento aeróbico e de resistência, além de estudos com amostras maiores, fornecerão mais orientação sobre este assunto. Estudos de acompanhamento de longo prazo, envolvendo grupos homogêneos de CPCA, e programas de exercícios padronizados podem fornecer mais informações sobre os benefícios potenciais da prática de exercícios físicos.
