# Supplementary material for: O Papel do Treinamento Físico na Melhoria da Qualidade de Vida para Cardiopatia Congênita Adulta: Revisão Sistemática
Source: Arq Bras Cardiol. 2024 Nov 22;121(12):e20240294. [Article in Portuguese] doi: 10.36660/abc.20240294 (PMC11634309; doi:10.36660/abc.20240294)
Supplement: Translation

# Translation: Examining the Role of Exercise Training in Enhancing Life for Adult Congenital Heart Disease: Systematic Review

**DOI:** 10.36660/abc.20240294i

**Authors:** Tugba Siyah, Naciye Vardar Yagli, Ilker Ertugrul, Hayrettin Hakan Aykan, Melda Saglam

**Affiliations:** 1 Turkey Hacettepe University, Faculty of Physiotherapy and Rehabilitation, Ankara – Turkey; 2 Turkey Hacettepe University, Faculty of Medicine, Department of Pediatric Cardiology, Ankara – Turkey

**Keywords:** Exercise, Congenital Heart Defects, Quality of Life, Endurance Training

## Abstract

**Background:**

Current guidelines advise exercise for most congenital heart disease patients (CHD). However, physical activity remains low in CHD individuals, with limited research on exercise’s effects in adults.

**Objectives:**

The aim of this study is to evaluate the safety and efficacy of exercise training on exercise capacity and quality of life in adult congenital heart disease (ACHD) patients.

**Methods:**

We searched PubMed/Medline, Cochrane Library, Web of Science, and Scopus through December 2022 for randomized controlled trials assessing aerobic and resistance training effects on exercise capacity and quality of life in ACHD. Out of 3,517 citations, ten eligible articles were included.

**Results:**

Meta-analysis of the included randomized controlled trials (286 participants) found no significant change in peak oxygen consumption or quality of life in ACHD with exercise training (pooled mean difference = 0.33 ml/kg/min [95% CI, -0.88 to 1.54 ml/kg/min]; p = 0.60; I^2^= 3%). However, the increase in maximum workload was significant (pooled mean difference = 8.86 watts [95% CI, 0.78 to 16.93], p = 0.03, I^2^ = 0%).

**Conclusions:**

Our review confirms that exercise training increases the maximum workload in ACHD patients. However, the lack of a standardized protocol among exercise interventions in this population may have contributed to the absence of a significant change in peak VO_2_ and quality of life observed in the conducted studies. The heterogeneity of exercise programs could be a contributing factor to the inconsistency of the results. In this context, the implementation of standardized exercise protocols in future research, particularly with larger sample sizes, is crucial to enhance the comparability of outcomes. Well-designed randomized controlled trials studying structured exercise training in ACHD patients will provide clearer insights.

## Introduction

Although the diagnostic and therapeutic possibilities for congenital heart disease (CHD) have improved significantly in recent years, CHD is one of the leading birth defects, with 13.3 million patients worldwide in 2019.^1,2^ Advances in interventional cardiology, congenital heart surgery, and CHD management have significantly reduced mortality from CHD and increased the population of patients with CHD reaching adulthood.^3^ These developments have rapidly made CHD a lifelong condition.^4^ The number of adults with CHD is expected to increase by 5% per year.^5^ With the increasing survival of patients with adult congenital heart disease (ACHD), functional health variables have become an important element in the assessment of treatment effects. In the past, CHD management was almost entirely the domain of pediatric cardiology, but it must now be continuous across the pediatric and adult healthcare systems.^4^ The growing ACHD population has increased the importance of management and treatment opportunities in adulthood. Therefore, research is ongoing to meet the various needs of the ACHD population, facilitate disease management, and develop relevant guidelines.

Exercise intolerance is a major problem for ACHD patients and significantly affects their quality of life. Most previous studies have been conducted with both pediatric and adult patients, with very few including only adults. There is no systematic review in the literature that includes studies only focused on adults. Thus, a systematic review examining the effects of exercise training on exercise capacity and quality of life in patients with ACHD may be beneficial in clinical practice. The purpose of this review was to examine randomized controlled trials evaluating the effectiveness and safety of aerobic and resistance exercise training programs in ACHD.

## Methods

This systematic review was conducted and reported in accordance with the PRISMA guidelines. The review protocol was registered in PROSPERO (International Prospective Register of Systematic Reviews; registration number CRD42022380143).

### Eligibility criteria

Randomized controlled trials that investigated the effects of aerobic and resistance exercise training in ACHD were included in this systematic review. Eligibility criteria included only individuals with CHD over the age of 18 years and involving aerobic exercise training, strength training, or a combination of both types of exercise training. Studies involving breathing exercises, oxygen therapy, and motivational techniques were excluded. The primary outcomes were exercise capacity (peak oxygen consumption [peakVO_2_]), maximal workload, and quality of life.

We searched for studies published in English between January 1980 and December 2022 in PubMed/Medline, the Cochrane Central Register of Controlled Trials, Web of Science, and Scopus. Keywords were checked in the Medical Subject Headings (MeSH) to develop a search strategy that was then used to obtain the titles and abstracts of potentially relevant studies. Two reviewers independently assessed the full text of the selected articles to verify whether they met the eligibility criteria. A data form was used to record data from the examined articles. The authors were contacted via email when any data confirmation or additional information was required.

Study characteristics (e.g., year of publication, study design), patient characteristics (e.g., age, diagnosis), exercise intervention data (e.g., aerobic, resistance, combined), and outcome data (e.g., incidence of adverse events, peakVO_2_, maximal workload, quality of life) were extracted from the included studies.

### Quality of evidence

The PEDro scale was used to assess the quality of evidence for this systematic review. The PEDro scale evaluates the methodological quality of the study with items such as inclusion/exclusion criteria, group allocation, and blinding.^6^

Seven of the included randomized controlled trials specified how randomization was performed. The randomization methods used in the studies included a computer-generated allocation sequence, randomization with sealed envelopes, and block randomization. Due to the nature of the interventions, it was impossible to blind participants and personnel. However, outcome measurements were blinded in three studies. All of the studies provided information about missing outcome data. There was no bias for selective reporting in any of the studies.

### Risk of bias assessment

Two independent reviewers assessed the risk of bias in the eligible studies using the Review Manager 5.4 (Cochrane, UK) tool for randomized controlled trials.

### Statistical analysis

For statistical analysis peakVO_2_, quality of life, and maximum workload data were recorded. The statistical analysis consisted of comparisons of peakVO_2_, quality of life, and maximum workload between the exercise and control groups (non-exercise, usual care) after the intervention. Pooled mean differences (MDs) with 95% CI and p values were obtained using a random effects model (DerSimonian-Laird). A p-value of 0.05 was considered significant. I^2^ values between 25% and 50% were accepted as an indicator of moderate heterogeneity. I^2^ values above 50% were accepted as an indicator of high heterogeneity. Review Manager 5.4 (Cochrane, UK) was used for the analysis of the extracted data.^7^

## Results

A total of 3517 publications were initially screened, and 83 publications that met the eligibility criteria were reviewed. Nineteen publications were excluded due to duplication. The full text of 64 articles was reviewed. Of these, 54 publications were excluded, and 10 publications were included in the study (Central Illustration). The minimum sample size was 10 and the maximum was 40. The average age of the participants ranged from 29 to 41 years.

Eight of the 10 studies involved aerobic exercise training. The study by Avila et al. involved combined aerobic and resistance exercise training,^8^ and the intervention conducted by Cordina et al. involved only resistance training. The included studies were published starting in 2003 and increased in number in the last 10 years.^9^ All of the control groups received standard care. Characteristics of the included studies are shown in Table 1.

**Table 1:** – Characteristics of the included studies

Reference	Publication Year	Patient diagnosis	Patient age (years)	Intervention	Control	Outcome Measures	Adverse events
Ávila et al.^8^	2016	ToF	35 ± 11	Combined aerobic and resistance training	Usual care (with exercise counseling)	PeakVO_2_ (ml/kg/min) BNP (pg/ml) Resting heart rate Metabolic equivalents (MET) Exercise duration	Mild dizziness during exercise training in 2 patients
Westhoff-Bleck et al.^15^	2013	TGA	29.3 ± 3.4	Aerobic exercise	Usual care	Peak VO_2_ (ml/kg/min) Borg rating of perceived exertion scale Exercise duration (min) Maximum workload (W) Kansas City Cardiomyopathy Questionnaire	No adverse event
Winter et al.^16^	2013	Systemic right ventricle, TGA	32 ± 11	Aerobic exercise	Usual care	PeakVO_2_ (ml/kg/min) NT-proBNP (ng/l) Short Form 36 item (SF-36)	No adverse event
Novaković et al.^10^	2018	ToF	39 ± 9	Aerobic exercise	Usual care (No supervised exercise)	PeakVO_2_ (ml/kg/min) Maximum workload (W) Metabolic equivalents (MET) NT-proBNP (ng/l) Short Form 36 item (SF-36)Adult Quality of Life (CHD-TAAQOL)	No adverse event
Sandberg et al.^13^	2018	Complex congenital heart disease (Pulmonary atresia, ToF, TGA)	29 ± 10	Aerobic exercise	Usual care	PeakVO_2_ (ml/kg/min) Maximum workload (W) EuroQol visual analog scale	In one case, exercise training was interrupted due to patient discomfort during the exercise training session and possible arrhythmia
van Dissel et al.^14^	2019	ToF, TGA, Fontan, Pulmonary atresia	40 ± 12	Aerobic exercise	Usual care	PeakVO_2_ (ml/kg/min) Anaerobic threshold (VE’/V’CO_2_ slope) Maximum workload (W) NT-proBNP (ng/l) Short Form 36 item (SF-36) Adult Quality of Life (CHD-TAAQOL	No adverse event
Opotowsky et al.^11^	2010	ToF with pulmonary stenosis or atresia or double-outlet right ventricle	41.1 ± 12.1	Aerobic exercise	Usual care	PeakVO_2_ (ml/kg/min) Maximum workload (W) Anaerobic threshold (VE’/V’CO2 slope) NT-proBNP (ng/l)	No adverse event
Rakhmawati et al.^17^	2020	Uncorrected ASD-PAH	37.5 ± 8.8 vs. 35.5 ± 10.4	Aerobic exercise	Usual care (subjects maintained regular activities and lifestyle)	6MWT distance (m) NT-proBNP (ng/l) EuroQol visual analog scale	No adverse event
Therrien et al.^12^	2003	ToF	35 ± 9.5 vs. 43.3 ± 73	Aerobic exercise	Usual care	PeakVO_2_ (ml/kg/min) Anaerobic threshold (VE’/V’CO_2_ slope)	Occasional premature ventricular and atrial beats in four patients during exercise testing
Cordina et al.^9^	2012	Fontan	32 ± 2	Resistance training	Usual lifestyle	PeakVO_2_ (ml/kg/min) Muscle strength	No adverse event

ToF: Tetralogy of Fallot; TGA: transposition of the great arteries; ASD: atrial septal defect; PAH: pulmonary arterial hypertension; peakVO_2_: peak oxygen consumption; bnp - brain natriuretic peptide; 6MWT: 6-minute walk test.

The exercise training interventions ranged from 10 to 24 weeks in duration. The most common frequency of exercise training was three times a week, and the duration of exercise sessions was between 30 and 60 minutes. Maximum Heart Rate (HRmax) and peakVO_2_ were the most preferred methods for determining exercise intensity in the included studies. Exercise intensity ranged from 60-80% of HRmax and 50-85% of peakVO_2_. Details pertaining to the interventions in the included reports are shown in Table 2.

**Table 2:** – Characteristics of the exercise training interventions included in the review

Reference	Type	Training duration (weeks)	Session duration (minutes)	Sessions per week	Intensity
Ávila et al.^8^	Combined aerobic and resistance training (jogging, rowing, swimming, and weight-bearing exercises)	12	60	1-2	70-80% HRmax
Westhoff-Bleck et al.^15^	Cycle ergometer	24	10-30	3-5	50% peakVO_2_
Winter et al.^16^	Step aerobic	10	42	3	75-90% HRmax
Novaković et al.^10^	Cycling and/or speed walking	12	42	2-3	60-80% HRmax
Sandberg et al.^13^	Cycle ergometer	12	42	3	Target HR at 75-80% intensity (Karvonen method) or Borg rating of perceived exertion 15-16
van Dissel et al.^14^	Sports of patient's preference (rowing, cycling, and ice skating)	24	45	3	80% HRmax
Opotowsky et al.^11^	Treadmill, cycling, or rowing	12	60	2	According to the participant’s response to exercise and perceived exertion rate
Rakhmawati et al.^17^	Treadmill and walking	12	30	3	60-70% HRmax
Therrien et al.^12^	Cycle ergometer and treadmill	12	30-50	3	60-85% peakVO_2_
Cordina et al.^9^	Chest press, lat (latissimus dorsi) pull-down, leg press, knee extension, knee flexion, hack calf, and seated calf	20	60	3	80% of the one-repetition maximum

HR: HRmax - Maximum Heart Rate; peakVO_2_ – Peak Oxygen Consumption.

### Outcomes

#### PeakVO2

Patients with ACHD who received the exercise training interventions had similar peakVO_2_ as patients who did not receive exercise training. In 8 studies, Cardiopulmonary Exercise Testing (CPET) with increasing workload was performed on the cycle ergometer. Only one study performed CPET on the treadmill. Analysis showed no statistically significant difference in improvement in peakVO_2_ in patients with ACHD between the exercise training intervention and control groups of the included randomized controlled trials (9 studies, n = 248: pooled MD: 0.33 ml/kg/min [95% CI: -0.88-1.54 ml/kg/min]; p = 0.60; I^2^= 3%) (Figure 1).^8-16^ Ávila et al. found that exercise training increased peakVO_2_ compared to baseline measurements, but there was no difference between the exercise and control group (intervention group: median 27.1 [interquartile range (IQR): 25.0-33.6] ml/kg/min vs. control group: 29 [IQR: 25.5- 31.0] ml/kg/min, p > 0.05).^8^ Cordina et al. associated exercise training with a significant increase in peakVO_2_ (Δ 183 ± 31 vs. 5 ± 39 ml/min, p = 0.02).^9^ Novakovic et al. compared interval training and continuous training with a control group and found that interval training improved peakVO_2_ while continuous training did not. There was no significant difference in peakVO_2_ values between the groups after the training (interval group: median 22.9 [IQR: 21.1-35.4] ml/kg/min, continuous group: 23.6 [IQR: 20.7-27.8] ml/kg/min, and control group: 21.5 [IQR: 19.4- 29.1] ml/kg/min).^10^ Similar to other studies, Opotowsky et al. found that peakVO_2_ value improved more in the exercise group compared to the control group (Δ 2.2 ml/kg/min, p = 0.002). However, the difference between groups after exercise training was not significant (intervention group: 16.4 ± 1.5 ml/kg/min vs. control group: 16.8 ± 2.3 ml/kg/min, p > 0.05).^11^

**Figure 1:** – Forest plot comparing exercise training vs. control; outcome: peakVO2 (ml/kg/min).

#### Maximum Workload

Maximum workload (in watts) was measured in 6 of the included randomized controlled trials (total n = 87 in the intervention group and n = 76 in the control group). Among these studies, the participants’ maximum workloads increased significantly after exercise training (6 studies: pooled MD: 8.86 [95% CI: 0.78-16.93] W, p = 0.03, I^2^= 0%) (Figure 2).^9-11,13-15^ Cordina et al. reported workload outcomes observed after 12 months of high-intensity resistance training (intervention vs. control group: 173 ± 15 vs. 149 ± 19 W, respectively, p = 0.01).^9^ In their comparison of interval training, continuous training, and the control group, Novaković et al. found that both interval and continuous training increased the maximum workload compared to baseline measurements (interval group: mean Δ 9 W, p = 0.002; continuous group: mean Δ 15 W, p = 0.003), but there was no difference between the groups.^10^ Opotowsky et al. found a nonsignificant improvement in peak work rate (Δ 8.1 W; p = 0.13, age-adjusted Δ 9.2 W; p = 0.16).^11^ In the Sandberg et al. study, post-intervention peak workload in the incremental CPET was higher in the intervention group compared to controls (median 170 [range: 90-240] vs. 140 [range: 110–200] W, respectively) and the change in workload was significantly greater for the intervention group (mean Δ 20 [range: –10-70] vs. Δ 0 [range: –20-15] W, respectively, p = 0.003).^13^ Similarly, Westhoff-Bleck et al. found that the maximum workload increased with exercise training (exercise group: 154.2 ± 46.4 W vs. control group: 140.6 ± 26.4 W).^15^ Contrary to the results of other studies, van Dissel et al. reported that the maximum workload was lower in the exercise training group than the control group. The reason for this result may be that the maximum workload of the control group was higher before training (exercise group: 183 ± 63 W vs. control group: 198 ± 55 W, p = 0.209).^14^

**Figure 2:** – Forest plot comparing exercise training vs. control; outcome: maximum workload (W).

#### Quality of life

Quality of life was assessed using the Kansas City Cardiomyopathy Questionnaire, The 36-Item Short Form Health Survey (SF-36), Dutch and Italian translations of the CHD TNO/AZL Adult Quality of Life (CHD-TAAQOL) Questionnaire, EuroQoL Vertical Visual Analogue Scale (EQ-VAS), and Minnesota Living with Heart Failure Questionnaire (MLHFQ). The most frequently used questionnaires were the EQ-VAS and SF-36. Participants showed no significant difference in EQ-VAS scores after exercise training (2 studies: MD: 3.64 points [95% CI: −1.77-9.05 points]; p = 0.19; I^2^= 0%) (Figure 3).^13,16,17^ In one of these studies, EQ-VAS scores in the exercise group improved incrementally from baseline to week 12 (mean Δ 15.5, p < 0.001).^17^ In contrast, Sandberg et al. reported no significant difference in the quality of life between the study groups (exercise group: mean Δ 0 [range: –21.0-25.0], control group: mean Δ 0 [range: –55.0-9.0], p = 0.42), which they attributed to the high quality of life in the study population before the intervention. Three studies evaluated quality of life with the SF-36, but data from only one study were available.^13^ Novakovic et al. observed that only the mental component of the SF-36 questionnaire improved compared to the baseline in the continuous exercise training group (mean Δ 7, p = 0.028). However, there were no other significant differences within or between the interval, continuous, and control groups.^10^

**Figure 3:** – Forest plot comparing exercise training vs. control; outcome: quality of life (EuroQoL EQ-VAS).

#### Adverse effects

None of the studies reported major exercise-related adverse events during exercise testing or training. Therrien et al. reported that occasional premature ventricular and atrial beats were recorded in four patients during exercise testing (two patients in baseline and two in follow-up assessment).^12^In the study by Sandberg et al., exercise training was interrupted due to patient discomfort and possible arrhythmia during the session in one case, but no arrhythmia was detected in the subsequent exercise test or Holter recording.^13^ Mild dizziness was reported during exercise training in two of the participants in another study.^8^

## Discussion

The results of this systematic review of randomized controlled trials of exercise interventions for patients with ACHD showed that exercise training increased maximum workload, however, more randomized controlled trials are needed to demonstrate the effects on exercise capacity as assessed by peakVO_2_ and quality of life (assessed by EQ-VAS and SF-36). No major adverse events were reported during the exercise intervention in any of the included studies, indicating that exercise training is safe for the ACHD population. This systematic review is the first study that investigates the impact of exercise training exclusively within research focused on the adult population.

Out of the studies included in this systematic review, seven studies demonstrated that aerobic exercise training contributed to improvements in peakVO_2_, while only two studies evaluating peakVO_2_ showed no improvement with aerobic exercise training. ACHD encompasses a wide variety of diseases, and the reviewed studies included patients with many different pathologies such as Tetralogy of Fallot, Transposition of the Great Arteries, Pulmonary Atresia Fontan, and Atrial Septal Defect. The inclusion of these conditions, which involve different pathologies, results in the formation of heterogeneous groups. It is known that peakVO_2_ is affected by many factors such as muscle oxygenation, oxygen-carrying capacity, endothelial function, forced expiratory volume in the first second (FEV_1_), diffusing capacity of the lung for carbon monoxide (D_LCO_), and muscle mass.^18,19^ Therefore, the inconsistent results and overall lack of a significant difference in peakVO_2_ with aerobic exercise training may be attributable to the heterogeneous study groups and the influence of multiple factors on peakVO_2_. Baseline peakVO_2_ values also impact post-exercise results. Although there was a change in peakVO_2_ with exercise training, lower baseline values in the exercise training group compared to the control group may be misleading in interpreting the results. In addition, insufficient sample size was noted in several studies. This situation and high standard deviations may be factors that affected the results. Furthermore, although treadmill and cycling were used most commonly, the exercise interventions in the included studies were quite heterogeneous. Ávila et al. included jogging, rowing, swimming, and weight-lifting exercises in their combined exercise training intervention, whereas van Dissel et al. used rowing, cycling, and ice-skating exercises in accordance with the patients’ preferences. Considering that the responses of different pathologies to exercise will differ in studies involving different CHDs, the exercise volume may have been insufficient for adults.^14^ Another recent systematic review and meta-analysis of studies involving patients with only tetralogy of Fallot evaluated the effect of exercise training on peakVO_2_.^20^ Although that meta-analysis included individuals with ACHD, the median age of the patients in the study was 7.4-43.3 years. The large age range can make it difficult to interpret the results for both the pediatric and adult populations.

Another important factor is the role of age in determining the appropriate exercise intensity and type. Including heterogeneous exercise volumes in studies precludes a clear understanding of the effect of exercise training on peakVO_2_. PeakVO_2_ increases in proportion to the increase in the exercise level, or workload. More energy per unit of time (power) is required to perform a heavier workload. Therefore, at greater workloads, VO_2_ increases proportionately.^21^ According to the results of the examined studies, a significant increase in workload was observed with aerobic exercise training.

In this review, we found that health-related quality of life after the exercise training intervention was similar in the intervention and control groups. Another review similarly led to the conclusion that any type of physical activity intervention (physical activity promotion, exercise training, and inspiratory muscle training) had little or no effect on health-related quality of life compared with standard care.^22^ One study indicated that a 12-week moderate- to high-intensity home-based cardiac physical activity program was safe and feasible for children with Fontan circulation and was associated with significant improvement in parent-reported health-related quality of life, but the program did not significantly improve the pediatric patients’ self-reported health-related quality of life.^23^ In contrast, Rhodes et al. observed improvements in health-related quality of life questionnaire scores in 15 patients with complex CHD after a 12-week hospital-based cardiac rehabilitation program and found that this improvement was sustained at 1-year follow-up.^24^ It should not be forgotten that in studies conducted in the pediatric population, families have high motivation, which may affect the results of training. Therefore, studies with a specific age range that includes children have very limited generalizability to the general population. In order to examine the effect of exercise training on health-related quality of life in the ACHD population, it is essential to evaluate health-related quality of life in adults only and observe the results of disease management with long-term follow-up. Quality of life can be influenced by numerous factors, such as physical well-being, financial situation, social life, and psychological and emotional state.^25^ For this reason, it would be more appropriate to examine data from more studies with further analyses to understand the effect of exercise training on quality of life. Also, instead of general quality-of-life questionnaires, instruments developed specifically for CHD may be better assessment tools.

Skeletal muscle dysfunction is known to be relatively common in people with ACHD.^26^ Sandberg et al. showed that adults with complex congenital heart lesions had impaired muscle function compared to patients with simple lesions and controls. Furthermore, the complexity of the heart lesion is an important determinant of limb muscle function.^27^ Targeted therapies to improve muscle weakness are recommended, as exercise limitations have been shown to be strong predictors of survival in the CHD population.^28^ Literature data pertaining to muscle strength assessment and strength training in adults with CHD are lacking. We believe that further studies in this area will be important because muscle strength is multifactorial and people with ACHD have muscle problems.^26,29^

No exercise-related major adverse events were reported in any of the studies. Traditionally, concerns about the safety of exercise training have resulted in many CHD patients being advised by their physicians not to participate in sports and other physical activities early in life, leading to parental or environmental overprotection and undue restrictions starting from childhood.^30^

Individuals with CHD have lower exercise capacity compared to healthy controls.^31^ This situation increases the importance of cardiac rehabilitation in patients with CHD. It has been suggested that the inclusion of cardiac rehabilitation and exercise training in disease management may be beneficial for ACHD patients with exercise intolerance.^4^ Although many studies have demonstrated the benefits of exercise training, it was reported that only 19% of people with CHD receive formal physical activity advice.^32^ This constitutes a risk factor for cardiovascular disease in later life.^33^ Therefore, exercise should be strongly encouraged for CHD patients without physical activity restrictions.^34^ The fact that the ACHD population is increasing and even outnumbing the pediatric CHD population necessitates an adult perspective on disease management and the planning of treatment programs to meet the needs of adult patients.

A small but growing body of literature suggests that in addition to routine medical therapy, patients with CHD who are clinically stable should be prescribed an individualized exercise education program commensurate to the characteristics of their condition after appropriate evaluation.^35^

### Limitations

There are some limitations to this systematic review. One of these is that few eligible trials evaluated resistance exercise and combined exercise as interventions and used exercise capacity and quality of life as outcome measures. Additionally, the included studies did not include long-term follow-up data, which would contribute to disease management after the exercise intervention.

## Conclusions

This systematic review shows that maximal workload increases with exercise training in the ACHD population. It also shows that there is little evidence from randomized trials to support the idea that exercise training improves exercise capacity and quality of life in ACHD. Therefore, well-designed randomized controlled trials of standardized exercise training, including aerobic and strength training, and studies with larger samples will provide further guidance on this subject. Long-term follow-up studies involving homogeneous CHD groups and standardized exercise programs may provide more information about the potential benefits of exercise training.
